# Neuroprotective effects of tannic acid in the postischemic brain via direct chelation of Zn^2+^

**DOI:** 10.1080/19768354.2022.2113915

**Published:** 2022-08-19

**Authors:** Seung Woo Kim, Da Bin Kim, Hong Seok Kim

**Affiliations:** a Department of Biomedical Sciences, College of Medicine, Inha University, Incheon, Korea; b Department of Molecular Medicine, College of Medicine, Inha University, Incheon, Korea

**Keywords:** Tannic acid, chelation, MCAO, neuroprotection

## Abstract

Tannic acid (TA) is a polyphenolic compound that exerts protective effects under pathological conditions. The diverse mechanisms of TA can exert beneficial anti-oxidative, anti-inflammatory, and anti-cancer effects. Herein, we reported that TA affords robust neuroprotection in an animal model of stroke (transient middle cerebral artery occlusion; tMCAO) and exhibits Zn^2+^-chelating and anti-oxidative effects in primary cortical neurons. Following tMCAO induction, intravenous administration of TA (5 mg/kg) suppressed infarct formation by 32.9 ± 16.2% when compared with tMCAO control animals, improving neurological deficits and motor function. We compared the chelation activity under several ionic conditions and observed that TA showed better Zn^2+^ chelation than Cu^2+^. Furthermore, TA markedly decreased lactate dehydrogenase release following acute Zn^2+^ treatment and subsequently reduced the expression of p67 (a cytosolic component of NADPH oxidase), indicating the potential mechanism underlying TA-mediated Zn^2+^ chelation and anti-oxidative effects in primary cortical neurons. These findings suggest that anti-Zn^2+^ toxicity and anti-oxidative effects participate in the TA-mediated neuroprotective effects in the postischemic brain.

## Introduction

Zn^2+^, an important trace element essential for human nutrition, acts as an intracellular secondary messenger that regulates several transcription factors and enzymes under various physiological and pathological conditions (Frederickson et al. 2005). Zn^2+^ has been detected in multiple brain regions, including the hippocampus, cerebral cortex, and hypothalamus (Donaldson et al. 1973). Zn^2+^ released from synaptic vesicles modulates receptors and ion channels that regulate synaptic transmission and neuronal excitability, thereby maintaining brain function. Acute exposure to Zn^2+^ increases neuronal cell death by inhibiting glycolysis and ATP production and elevating levels of reactive oxygen species (ROS) by activating NADPH oxidase in neurons (Noh and Koh 2000; Sheline et al. 2000). Moreover, excessive Zn^2+^ intake activates poly ADP ribosyl polymerase-1 (PARP-1) and induces neuronal cell death via NAD depletion (Ha and Snyder 1999; Kim et al. 2002; Sheline et al. 2003). The membrane-permeable zinc chelator N,N,N’,N'-tetrakis (2-pyridylmethyl) ethylenediamine (TPEN) was found to reduce infarct formation, improve neurological deficits, and decrease Zn^2+^ accumulation, further suppressing PARP-1 activation (Zhao et al. 2014)

Tannins, categorized into hydrolyzable and non-hydrolyzable (condensed) tannins, are widespread in fruits, cereals, legumes, herbs, green tea, red wine, and vegetables (Chung et al. 1998). Proanthocyanidin (condensed tannin) reportedly exerts cardioprotective effects against ischemia/reperfusion injury by scavenging ROS and inhibiting pro-apoptotic transcription factors and genes *JNK*-*1* and *c-Jun* (Sato et al. 1999). Punicalagin (hydrolyzable tannin) can suppress cerebral ischemic injury by inducing anti-inflammatory effects (Yaidikar and Thakur 2015). Furthermore, geraniin (hydrolyzable tannin) can upregulate nuclear factor-E2 related factor 2 (Nrf-2), which contributes to enhanced anti-oxidative enzyme expression (Wang et al., 2015). Hydrolyzable tannins such as ellagitannins and gallotannins, comprising a general structural motif of galloyl groups, exhibit anti-oxidative and neuroprotective effects (Chung et al. 1998). Moreover, hydrolyzable tannins act as chelators to regulate enzyme activity (Chen et al. 2019). Tannic acid (TA) is a water-soluble polyphenol compound and commercial tannin. TA reportedly exerts anti-carcinogenic, anti-oxidative, and anti-inflammatory effects. TA can attenuate skin cancer induced by 7,12-dimethylbenz[a]anthracene (DMBA) and croton oil by suppressing oxidative and inflammatory responses (Majed et al. 2015). Pretreatment with TA was shown to suppress infarct formation and improve neurological deficits in the middle cerebral artery occlusion (MCAO) stroke model. Depletion of antioxidant enzyme activity was markedly ameliorated during infarct progression in the TA-pretreated MCAO group (Ashafaq et al. 2016). Intraperitoneal administration of TA can upregulate Nrf2, which activates antioxidant enzymes, including heme oxygenase-1 (HO-1) and superoxide dismutase (SOD), in animal models of traumatic brain injury and nephrotoxicity (Jin et al. 2020; Salman et al. 2020). In addition, TA can attenuate ultraviolet (UV)-induced inflammation, which induces interleukin (IL)−6 production and phosphorylates STAT3 in the epithelial cells (Chou et al. 2012). Moreover, TA could inhibit NF-κB signaling in atopic dermatitis (AD) NC/Nga mice (Karuppagounder et al. 2015). Oral administration of TA afforded marked protection against oxidative stress, given that TA acts as a scavenger and chelator in iron-dextran-induced hepatotoxicity (Basu et al. 2018).

In the present study, we revealed that TA exerted robust neuroprotection against ischemic stroke, markedly suppressing infarct formation and improving behavioral deficits. In addition, we found that TA directly chelated Zn^2+^ and inhibited Zn^2+^-mediated ROS production, thereby suppressing neuronal cell death. Therefore, TA provides profound neuroprotection in the postischemic brain, and this neuroprotective effect is partially attributed to its role as a chelator.

## Materials and methods

### Surgical procedures for MCA occlusion

In the present study, animal comfort and pain minimization were carefully considered. The animal research protocol was reviewed and approved by the Inha University-Institutional Animal Care and Use Committee prior to study initiation at Inha University. This study was performed in accordance with the Guide for the Care and Use of Laboratory Animals published by the National Institute of Health (2010) and the ARRIVE (Animal Research: Reporting In Vivo Experiments) guidelines. Male Sprague–Dawley (SD) rats (230-250 g, 8-week-old) were purchased from Orient Bio Inc. (Gyeonggi, South Korea) and housed under a standard light–dark cycle. Food and water were provided *ad libitum*. MCAO was performed as previously described (Yu et al. 2021). Briefly, SD rats (250-300 g) were anesthetized with isoflurane (2% induction, 1.5% maintenance) using an oxygen/nitrous oxide (30/70%) mixture. MCAO was performed for 60 min using nylon suture (4-0; AILEE, Busan, South Korea), followed by reperfusion. A laser Doppler flowmeter (Periflux System 5000; Perimed, Jarfalla, Sweden) was used for real-time monitoring of regional cerebral blood flow. During surgery, the rectal temperature of the rat was maintained within 37.0 ± 0.5°C using a heat pad.

### TA injection to MCAO-operated rats

TA (1, 2.5, or 5 mg/kg) was dissolved in saline (50 μl) and administered intravenously (i.v.) 1 h after MCAO. Animals were randomly divided into three groups as follows: a sham group (n = 3), including animals who underwent surgery without MCAO induction; an MCAO group, including treatment-naive MCAO controls (n = 6); an MCAO + TA group, including TA-administered MCAO rats (n = 21).

### Infarct volume assessment

To measure brain infarct volume, brains were dissected coronally into 2 mm slices using a metallic brain matrix (RBM-40,000, ASI, Springville, UT, USA) 2 days post-MCAO. Brain slices were quickly incubated in saline containing 2, 3, 5-triphenyl tetrazolium chloride (TTC, 2%) at 37°C for 15 min and fixed with 4% paraformaldehyde (PFA). The infarcted brain tissue areas were analyzed using the Scion Image program (Scion Image program, Frederick, MD, USA). To correct for edema and shrinkage, the infarct volume was calculated using the following formula: (contralateral hemisphere volume × measured injury volume/ipsilateral hemisphere volume).

### Evaluation of modified neurological severity scores

Modified neurological severity scores (mNSS) were used to evaluate neurological deficits 2 days post-MCAO. The total mNSS score was 18 points (normal, 0; maximal deficit, 18) and consisted of sensory, motor, reflex, and balance tests (Chen et al. 2001).

### Wire hanging test

The wire hanging test, which measures forelimb strength and grasping ability, was performed as previously described (Rakhunde et al. 2014). Briefly, SD rats were suspended by their forelimbs on a steel wire (50 cm long, 2 mm diameter), and the latency to fall was measured using a stopwatch until a cut-off time of 60 s was reached.

### Rotarod test

One day before MCAO, the SD rats were trained on a rotarod apparatus (Daejon Instruments, Seoul, Korea) at a speed of 3 rpm until they could remain on the rotating spindle for 180 s. Two days post-MCAO, the latency times on the spindle were measured at spindle speeds of 5 and 10 rpm, with a 1 h rest period after testing at 5 rpm.

### Measurement of zn^2+^ and cu^2+^ chelating activity

The Zn^2+^ and Cu^2+^ chelating activities were determined using Zincon (Merck, Darmstadt, Germany). All samples (1 ml) were prepared in borate buffer (50 mM, pH 9.0) containing Zincon (40 μM) and urea (8 M) and subsequently incubated at room temperature for 5 min. Absorbance was measured at 615 nm. The percentage of Zn^2+^ and Cu^2+^ chelation activities was calculated using the following equation: chelation (%) = (A0 – A1)/A0  100. A0 = Zn^2+^ or Cu^2+^ absorbance, and A1 = Zn^2+^+TA or Cu^2+^+TA absorbance.

### Cortical primary neuron culture

Primary cortical cells were obtained from rat cortices (E15.5) and cultured as previously described (Kim et al. 2020). Cortical cells (4×10^5^ cells per well) were plated on poly-d-lysine (100 μg/ml)-and laminin (100 μg/ml)-coated plates. The cultured cortical cells were maintained without antibiotics in modified Eagle’s medium (MEM) containing 5% fetal bovine serum (FBS), 5% horse serum, 21 mM glucose, and 2 mM glutamine. On day in vitro (DIV) 7, cytosine arabinofuranoside (10 μΜ) was added and maintained for two days to halt microglial growth. FBS and glutamine were not supplemented from DIV 7, and the medium was replaced every other day after DIV 7. Cultures were used on DIV 12-14. Primary cortical cells were treated with 300 μM Zn^2+^ for 15 min in HCSS (HEPES-controlled salt solution) and then incubated with 21 mM glucose-containing MEM.

### Immunoblotting

Briefly, cortical cultures were washed twice with cold phosphate-buffered saline (PBS) and lysed in RIPA lysis buffer (50 mM Tris-HCl [pH7.4], 1% NP-40, 0.25% sodium deoxycholate, and 150 mM NaCl) containing protease and phosphatase inhibitor cocktail as previously described (Xiao et al. 2021). Lysates were centrifuged for 15 min at 14,000 rpm at 4°C, and supernatants were loaded onto 6-12% sodium dodecyl sulfate-polyacrylamide gel electrophoresis (SDS-PAGE) gels. Primary antibodies for anti-p67 (Abcam, Cambridge, UK) and anti-α-tubulin (Santa Cruz Biotechnology, Santa Cruz, CA, USA) were diluted to 1:2000. Primary antibodies were detected using a chemiluminescence kit (Merck Millipore, Darmstadt, Germany) with horseradish peroxidase-conjugated secondary antibody (1:4000; Merck Millipore, Darmstadt, Germany).

### ROS quantification

ROS quantification in the cortical cultures was performed using the 5-(and-6)-chloromethyl-2’, 7’-dichlorodihydrofluorescein diacetate assay (CM-H2DCFDA; Invitrogen). Briefly, cortical cultures were incubated for 30 min in MEM containing 5 μM CM-H2DCFDA and washed twice with PBS. ROS fluorescence was visualized under a Zeiss microscope (Oberkochen, Germany), and the intensities were measured using ImageJ software (http://rsbweb.nih.gov/ij/).

### Statistical analysis

Statistical analysis was performed using analysis of variance followed by the Newman–Keuls test. The analysis was performed using the GraphPad PRISM software 5.0 (GraphPad Software, Inc., La Jolla, CA, USA). Results are presented as the mean ± standard error of the mean (SEM), and statistical significance was set at α = 0.05.

## Results

### TA suppressed infarct formation in the postischemic brain

**Figure 1:**
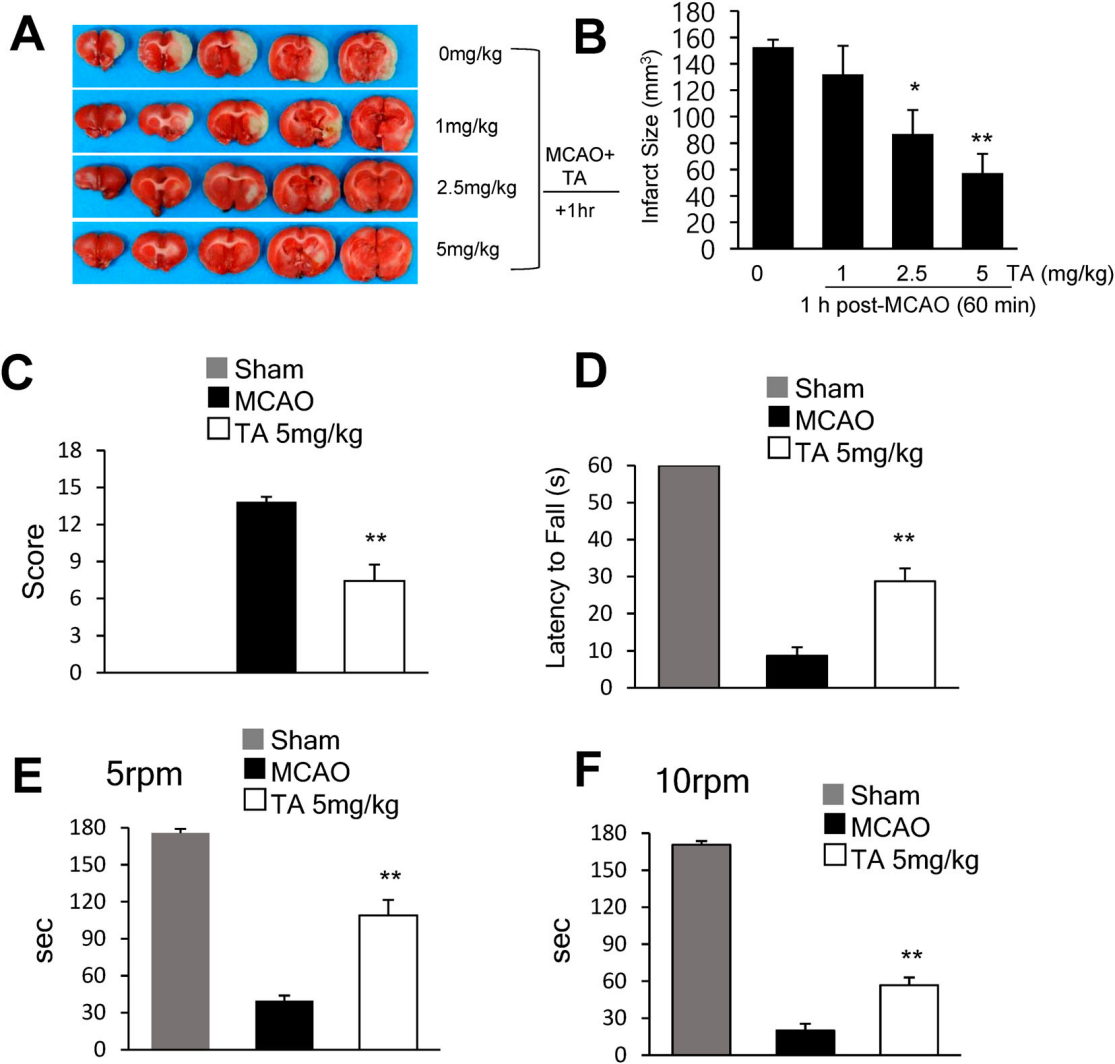
. Neuroprotective effects of tannic acid in the postischemic brain. (A) TA (1, 2.5, or 5 mg/kg, i.v.) was administered 1 h after MCAO, and mean infarct volume was measured by TTC staining. Representative images of infarctions in coronal brain slices are shown (A), and quantitative results are presented as the mean ± standard error of the mean (SEM) (n = 6-7) (B). (C-F) TA (5 mg/kg, i.v.) was administered 1 h after MCAO. (C) Neurological deficits were evaluated using mNSS 2 days after MCAO. (D) Wire hanging test was performed to assess motor impairment 2 days after MCAO. (E, F). The rotarod test was performed 2 days after MCAO. Latency time was recorded at a spindle speed of 5 and 10 rpm. Results are presented as the mean ± SEM (n = 6–7). * *p* < 0.05, ** *p* < 0.01 *vs*. MCAO group. MCAO, middle cerebral artery occlusion; mNSS, modified neurological severity scores; i.v., intravenously; TA, tannic acid.

To investigate the neuroprotective effects of TA in the postischemic brain, 1, 2.5, or 5 mg/kg TA was administered (i.v.) 1 h post-MCAO, and infarct volumes were measured 2 days post-MCAO. Administration of 2.5 or 5 mg/kg of TA (i.v.) at 1 h post-MCAO reduced the mean infarct volume to 57.6 ± 14.1% (n = 7, *p* < 0.05) and 32.9 ± 16.2% (n = 7, *p* < 0.01) when compared with the MCAO control group, respectively (Figure 1B). On administering 5 mg/kg TA 1 h post-MCAO, the mean mNSS at 2 days post-MCAO was 7.4 ± 1.3 (n = 4, *p* < 0.01); this was significantly lower than that in the vehicle-treated MCAO control group (13.8 ± 0.4) (n = 4) (Figure 1C). Motor function was evaluated using the wire hanging (Figure 1D) and rotarod (Figure 1E, F) tests. In the wire hanging test, the latency to fall was measured two days post-MCAO. The mean hanging time of the TA-treated MCAO group (28.7 ± 3.5 s) (n = 7) was significantly greater than that of the vehicle-treated MCAO control group (8.7 ± 2.3 s) (n = 6, *p* < 0.05) (Figure 1D). In the rotarod test, the latency to fall was measured at 5 and 10 rpm 2 days post-MCAO. At 5 rpm, the TA-treated MCAO group (98.6 ± 12.5 s) (n = 7, *p* < 0.05) showed a longer mean time spent on the rotarod than the vehicle-treated MCAO control (43.3 ± 4.1 s) (n = 6) (Figure 1E). Similarly, at 10 rpm, the mean time was significantly longer in the TA-treated MCAO group (56.7 ± 6.3 s) (n = 7, *p* < 0.05) than that in the vehicle-treated MCAO control group (20 ± 5.5 s) (n = 6) (Figure 1F). These results indicated that TA showed neuroprotective potency and improved behavioral outcomes.

### TA suppressed zn^2+^-induced neuronal cell death in primary cortical culture

**Figure 2:**
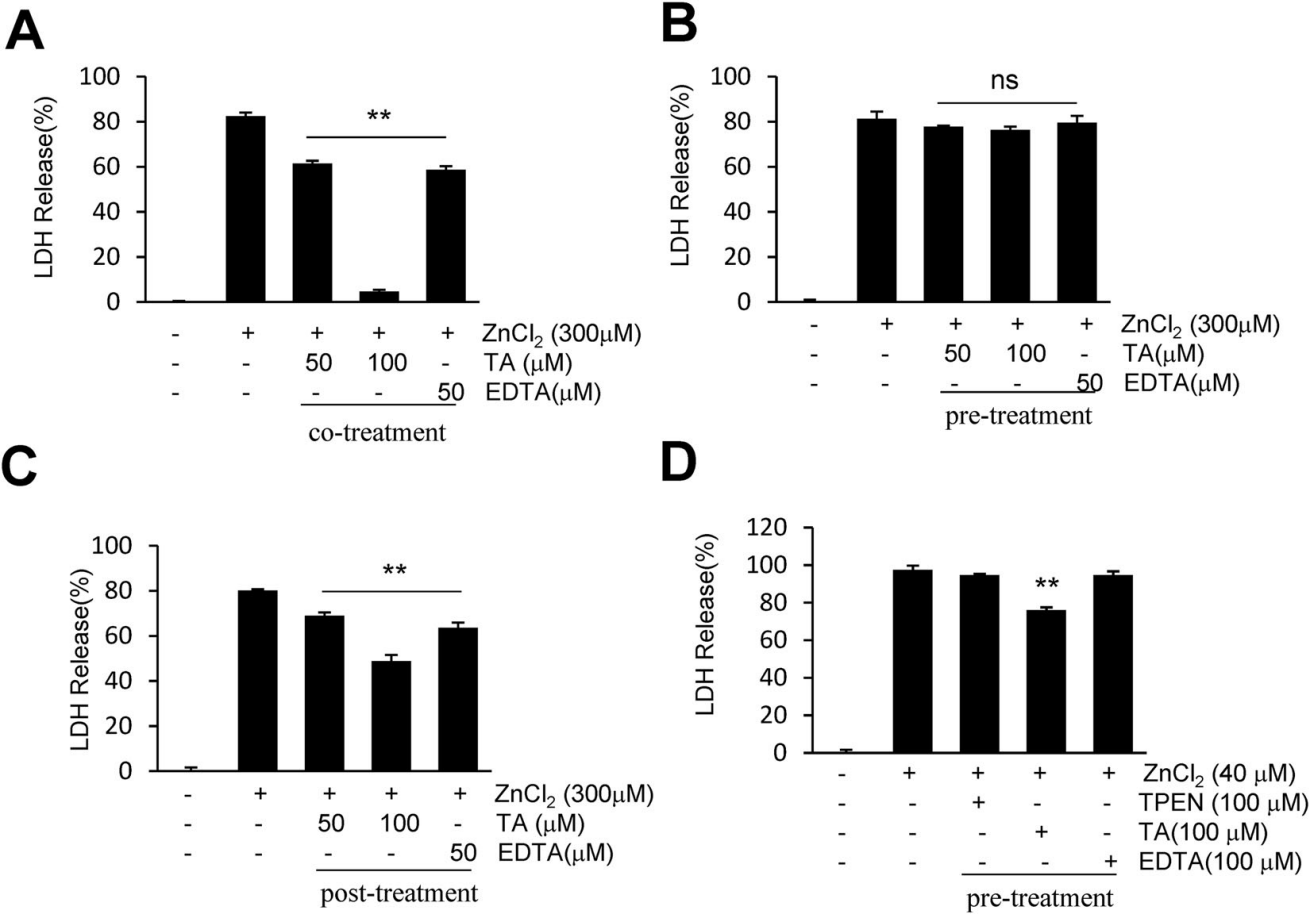
. Tannic acid suppresses LDH release in Zn^2+^-treated cortical neurons. (A-C) Primary cortical neurons were co-, pre-, or post-treated with TA (50 or 100 μM) or EDTA (50 μM) for 15 min in the presence or absence of 300 μM of Zn^2+^. (D) Primary cortical neurons were pretreated with TA (100 μM), EDTA (100 μM), or TPEN (100 μM) for 30 min, followed by Zn^2+^ treatment for 24 h. Neuronal cell death was measured using the LDH assay. Results are presented as the mean ± standard error of the mean (SEM) (n = 4) ** *p* < 0.01 *vs*. Zn^2+^-treated controls. LDH, lactate dehydrogenase; TA, tannic acid.

To explore the neuroprotective mechanisms of TA, we examined whether TA suppresses lactate dehydrogenase (LDH) release during Zn^2+^-induced neuronal cell death. An LDH cytotoxicity assay was conducted to examine the cytotoxic potential of Zn^2+^ on primary cortical neurons (Fig. S1). To determine the characteristics of TA, primary cortical neurons underwent pre-, co-, and post-treatment with TA. Following acute incubation with Zn^2+^, co-treatment with TA (100 μM) or EDTA (50 μM) suppressed LDH release (4.6 ± 1.6% or 58.6 ± 3.2%, respectively) when compared with Zn^2+^ only-treated neurons (Figure 2A). Similarly, post-treatment with TA (100 μM) or EDTA (50 μM) suppressed LDH release (48.8 ± 5.4% or 63.5 ± 4.7%, respectively) when compared with Zn^2+^ only-treated neurons (Figure 2C). However, pretreatment with TA or EDTA failed to afford protection against Zn^2+^-induced neuronal cell death (Figure 2B). Following chronic incubation with Zn^2+^, TA reduced LDH release when compared with the Zn^2+^ only-treated neurons (76 ± 2.9%), whereas EDTA (a cell membrane-impermeable chelator) or TPEN (a cell membrane-permeable chelator) afforded no protection after pretreatment with Zn^2+^ (Figure 2D). These results indicated that TA suppressed the LDH release to counteract Zn^2+^-induced neuronal toxicity.

### TA directly chelated zn^2+^ under cell-free conditions

**Figure 3:**
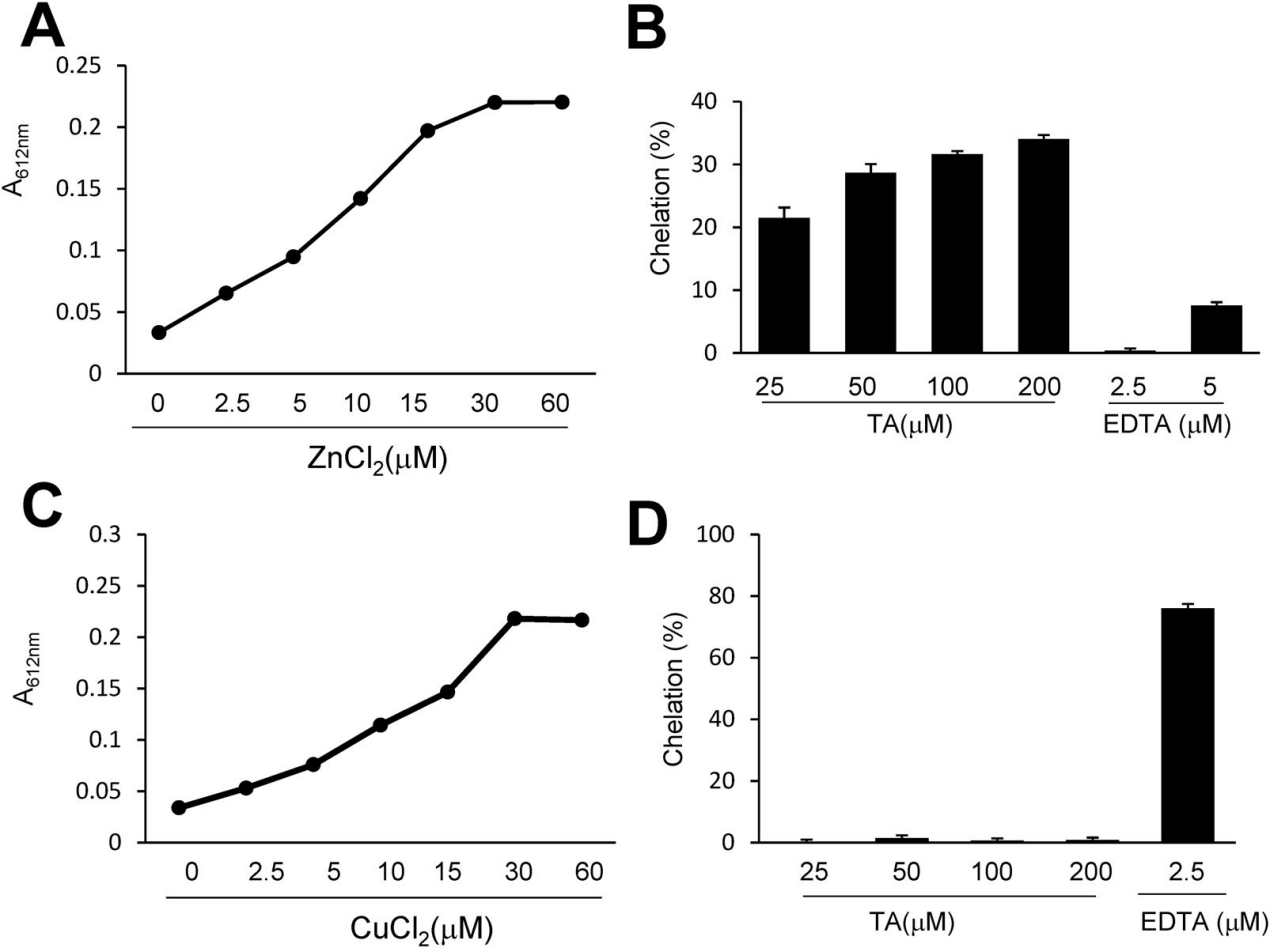
. Measurement of the chelation activity of tannic acid using Zincon. (A, C) Zincon (40 μM) was exposed to Zn^2+^ or Cu^2+^ at various concentrations (2.5, 5, 10, 15, 30, or 60). (B, D) TA (25, 50, 100, or 200 μM) or EDTA (2.5 or 5 μM) was incubated with Zn^2+^ or Cu^2+^ (30 μM) in the presence of Zincon (40 μM). The absorbance was measured at 620 nm. Results are presented as the mean ± standard error of the mean (SEM) (n = 4). TA, tannic acid.

To measure the Zn^2+^ or Cu^2+^ chelation efficiency of TA, we used Zincon, a colorimetric indicator of Cu^2+^ and Zn^2+^. Zn^2+^ or Cu^2+^ (0, 2.5, 5, 10, 15, 30, and 60 μM) was incubated with Zincon for 5 min, and the absorbance (615 nm) was recorded. As shown in Figure 3A and C, the absorbance of Zn^2+^- or Cu^2+^-Zincon complexes peaked at 30 μM in the borate buffer. TA (25, 50, 100, and 200 μM) was incubated with 30 μM Zn^2+^ for 5 min in the presence of Zincon (40 μM) and the color change was measured. At all examined concentrations, TA chelated Zn^2+^, but not Cu^2+^, in a concentration-dependent manner (Figure 3B, D). On incubating 100 μM TA with Zn^2+^ (30 μM) for 5 min, Zn^2+^ chelation increased by 31.6 ± 0.5%. However, 100 μM of TA did not chelate Cu^2+^ (30 μ µM). These results implied that TA selectively chelated Zn^2+^ in the presence of Zincon.

### TA suppressed ROS generation via zn^2+^ chelation

**Figure 4:**
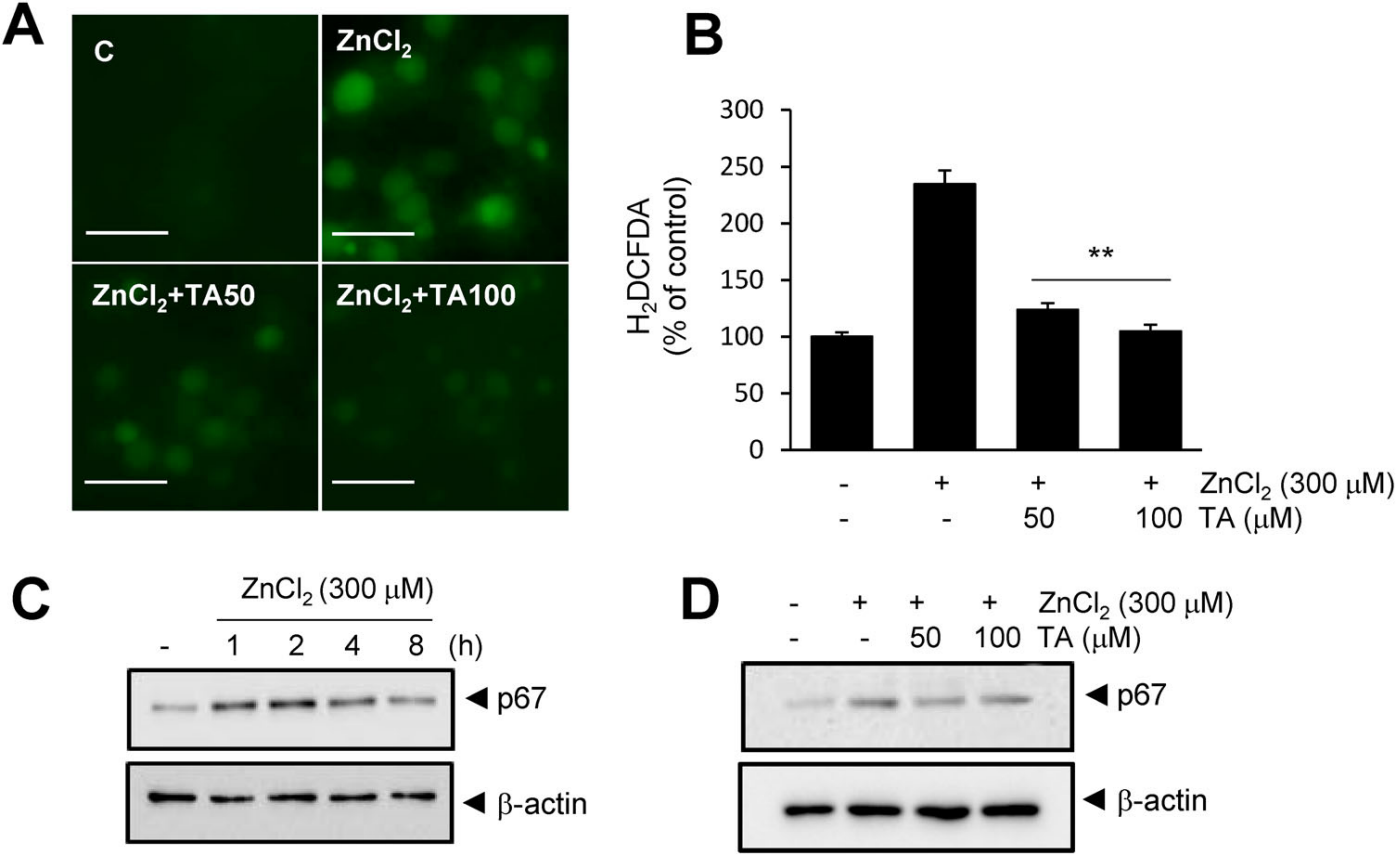
. Tannic acid suppresses ROS generation in primary cortical neurons. (A, B) Primary cortical neurons were treated with Zn^2+^ (300 μM) and TA (50 or 100 μM) for 15 min. Intracellular ROS levels were measured using H2DCFDA at 4 h, and fluorescence images of intracellular ROS were obtained using a live cell imaging microscope. Results are presented as the mean ± standard error of the mean (SEM) (n = 9) ** *p* < 0.01 *vs*. Zn^2+^-treated controls. (C) p67 protein level was examined by immunoblotting after treatment with Zn^2+^ (300 μM) for 1, 2, 4, and 8 h. (D) The effect of TA (50 and 100 μM) on p67 elevation was examined by immunoblotting after 2 h of Zn^2+^ (300 μM) treatment. β-actin was used as a loading control. Scale bar = 20 μm. ROS, reactive oxygen species; TA, tannic acid.

Finally, we investigated whether TA-mediated Zn^2+^ chelation confers an anti-oxidative function. ROS generation was examined in primary neuronal cells. TA suppressed Zn^2+-^induced ROS generation in a dose-dependent manner, and 50 or 100 μM TA suppressed ROS generation to basal levels (Figure 4A, B). Additionally, TA suppressed p67 (NADPH oxidase subunit) expression in Zn^2+^-treated cortical neurons (Figure 4D). In addition, TA blocked NMDA-induced neuronal cell death, and 50 μM TA reduced LDH release to 52.8 ± 6% in NMDA controls (Fig. S2A). Treatment with 50 or 100 μM TA reduced NMDA-induced ROS generation to 51.2 ± 4.6 and 71.5 ± 5.9%, respectively (Fig. S2B, C). Collectively, these results indicated that TA exerts its anti-oxidative effect via chelation and inhibition of p67 expression.

## Discussion

The findings of the present study suggest that TA could reduce infarct formation and improve neurological deficits in patients with postischemic stroke. Herein, TA exerted neuroprotective effects via Zn^2+^ chelation and reduced ROS generation. Ashafaq et al. have suggested the neuroprotective effects of TA in the MCAO model; however, detailed mechanisms explaining TA-mediated inhibition of inflammation or oxidative stress are lacking. A protective mechanism of TA by inhibiting NF-κB has been previously suggested in nephrotoxicity and cardiotoxicity (Jin et al., 2020; Xue et al. 2020); however, the TA mechanism underlying these effects remains unclear. We speculated that the neuroprotective effects of TA were mainly derived from its chelating effect, given that TA is a water-soluble polyphenol with an 8 gallic acid group and acts as a metal chelator (Strlic et al. 2002). Chelation therapy is a medical treatment used to reduce the toxic effects of metals. Chelating agents easily bind to metal ions to form complex structures that inhibit toxic effects by excreting the formed complexes from intracellular or extracellular spaces. Cerebral ischemia induces substantial intracellular Zn^2+^ accumulation, contributing to brain damage (Koh et al. 1996). Ca-EDTA, a high-affinity zinc chelator, can reduce neuronal cell death and attenuate the mitochondrial release of cytochrome C and Caspase-3 activation (Calderone et al. 2004). Furthermore, TPEN, a membrane-permeable zinc chelator, was found to reduce Zn^2+^ accumulation in cerebral ischemia animal models by inhibiting PARP-1 activation (Zhao et al. 2014). 2, 2’–ipyridyl, a lipophilic iron chelator, can attenuate apoptotic cell death in photothrombosis-induced focal ischemia (Hoecke et al., 2005). In particular, we found that TA exhibited a strong Zn^2+^ chelating effect, which contributed to the suppression of infarct formation and improvement of neurological impairment.

Reportedly, TA exerts anti-oxidative effects by regulating NF-κB and Nrf-2 activity (Jin et al. 2020; Salman et al. 2020). TA suppresses lipid oxidation, regulates anti-oxidative enzymes in aluminum oxidative neurotoxicity (Tüzmen et al. 2015). In the present study, we found that the anti-oxidative effect of TA was related to Zn^2+^ chelation (Figure 3B), which subsequently suppressed ROS production and expression of the NADPH oxidase subunit (Figure 4D). This TA-induced Zn^2+^ chelation is speculated to be, at least partially, responsible for the neuroprotective effects in the postischemic brain. Chelation of intracellular Zn^2+^ can suppress brain damage. The membrane-permeable zinc chelator TPEN plays a role in reducing infarct volume, improving motor function, and suppressing cerebral ischemia-induced blood-barrier damage in the postischemic brain (Zhao et al. 2014; Wang et al., 2015; Qi et al. 2016). Interestingly, our study revealed that TA selectively chelated Zn^2+^ but not Cu^2+^ (Figure 3). However, tannin fractions from walnuts exhibit stronger Cu^2+^ chelation than Zn^2+^ chelation (Karamać 2009). It is speculated that Zincon (a colorimetric indicator for Zn^2+^ and Cu^2+^) does not undergo chelation with TA, given its higher affinity for Cu^2+^ than TA (Figure 3D).

To determine the pharmacological characteristics of TA, we performed pre-, post-, and co-treatment with TA in a Zn^2+^-induced neuronal cell death model (Figure 2). We noted the characteristic extracellular chelator activity of TA in acute Zn^2+^ toxicity, similar to that of EDTA. In chronic Zn^2+^ toxicity, pretreatment with TA reduced LDH release; however, pretreatment with EDTA or TPEN failed to exhibit this effect (Figure 2D). Given that TA can activate the Nrf2 pathway, pretreatment with TA could afford protection against chronic Zn^2+^ toxicity. Interestingly, the duration of TA pretreatment and ZnCl_2_ treatment are important factors governing the activation of Nrf2 signaling and protection against Zn^2+^ toxicity. In the chronic Zn^2+^ treatment, pretreatment with TA was performed for 15 min longer than that in acute Zn^2+^ treatment (Figure 2B, D). NMDA receptor activity induces elevated Zn^2+^ levels and ROS generation (Bossy-Wetzel et al. 2004). The activation of NMDA receptors leads to superoxide generation, mainly induced by NADPH oxidase (Brennan et al. 2009), and excitotoxicity, which is mediated by PARP-1 (Mandir et al. 2000). NMDA receptor-induced calcium influx produces prolonged elevation of intracellular Ca^2+^ concentrations and triggers signaling pathways (Dingledine et al. 1999), wherein TA can chelate Ca^2+^. However, we only demonstrated that TA could inhibit NMDA-induced LDH release and ROS generation in cortical neurons, and further experiments are warranted (Fig. S2). In addition, we used oxygen/glucose deprivation (OGD), which mimics *ex vivo* ischemic stroke. The OGD-induced Zn^2+^ influx was reduced by co-treatment with TA or TPEN (Fig. S3). TA appears to exert a chelating effect in extracellular and intracellular environments and activates the Nrf2 pathway (Jin et al. 2020; Salman et al. 2020).

In summary, TA exerts neuroprotective effects in the postischemic brain. Our findings suggest that TA-mediated chelation induces anti-oxidative and anti-Zn^2+^ toxicity effects.
